# Green Tea Catechin Association with Ultraviolet Radiation-Induced Erythema: A Systematic Review and Meta-Analysis

**DOI:** 10.3390/molecules26123702

**Published:** 2021-06-17

**Authors:** Mahendra P. Kapoor, Masaaki Sugita, Yoshitaka Fukuzawa, Derek Timm, Makoto Ozeki, Tsutomu Okubo

**Affiliations:** 1 Nutrition Division, Taiyo Kagaku Co. Ltd., 1-3 Takaramachi, Yokkaichi 510-0844, Japan; mozeki@taiyokagaku.co.jp (M.O.); tohkubo@taiyokagaku.co.jp (T.O.); 2 Faculty of Sport Science, Nippon Sport Science University, 7-1-1 Fukusawa, Setagaya-Ku, Tokyo 158-8508, Japan; m-sugita@nittai.ac.jp; 3 Preemptive and Integrative Medicine Center, Aichi Medical University Hospital, 1-1 Karimata, Yazako, Nagakute 480-1195, Japan; yofuku@aichi-med-u.ac.jp; 4 Taiyo International Inc., 5960 Golden Hills Dr, Minneapolis, MN 55416, USA; derek@taiyoint.com

**Keywords:** green tea catechins, meta-analysis, ultraviolet radiation, skin, erythema (sunburn)

## Abstract

Catechins are a part of the chemical family of flavonoids, a naturally occurring antioxidant, and a secondary metabolite in certain plants. Green tea catechins are well recognized for their essential anti-inflammatory, photo-protective, antioxidant, and chemo-preventive functions. Ultraviolet radiation is a principal cause of damage to the skin. Studies observed that regular intake of green tea catechins increased the minimal dose of radiation required to induce erythema. The objectives of this systematic review and meta-analysis are to determine the effectiveness of green tea catechins in cutaneous erythema and elucidate whether green tea catechin consumption protects against erythema (sunburn) inflammation. A comprehensive literature search was conducted to identify the relevant studies. Two researchers carried out independent screening, data extraction, and quality assessment according to the guidelines of Preferred Reporting Items for Systematic Reviews and Meta-Analysis (PRISMA). The pooled effect of green tea catechins on protection against erythema was assessed using approaches fixed-effects or random-effects model to quantify the effectiveness of green tea catechins in the erythema dose–response. Studies not be included in meta-analyses were summarized narratively. Six randomized controlled studies of enrolled studies regularly administrated green tea catechins orally for 6 to 12 weeks involving healthy volunteers comprising a total of 100 participants were included in the analysis. The results revealed green tea catechins have favorable protection against erythema inflammation even at increased minimal erythema dose (MED) of ultraviolet radiation. Meta-analysis results confirm oral supplementation of green tea catechins is highly effective at low-intensity ultraviolet radiation-induced erythema response (MED range; 1.25–1.30) compared to placebo, showing a significant pooling difference (*p* = 0.002) in erythema index (SMD: −0.35; 95% CI, −0.57 to −0.13; I^2^ = 4%, *p* = 0.40) in the random-effects model. The pro-inflammatory signaling pathways through oral supplementation with green tea catechins are an attractive strategy for photo-protection in healthy human subjects and could represent a complementary approach to topical sunscreens. Therefore, studies that involved green tea catechin in topical applications to human subjects were also evaluated separately, and their meta-analysis is presented as a reference. The evidence indicates that regular green tea catechin supplementation is associated with protection against UV-induced damage due to erythema inflammation.

## 1. Introduction

Ultraviolet radiation in sunlight is the primary etiological element in major skin-related diseases. Vasodilatation of the dermal vasculature is one of the complex features of skin observing ultraviolet radiation exposure. Acute and repeated exposures of ultraviolet radiation (295–400 nm) induce erythema, i.e., redness of the skin [1,2,3], and result in changes in gene expression, generation of cytokines, upregulation of pro-inflammatory lipid mediators, including the generation of reactive oxygen species (ROS), photosensitivity disorders and premature photo-aging of skin [4,5,6,7]. Erythema reaction is usually known to be induced by increased eicosanoids prostaglandin E_2_ (PGE_2_), 12-hydoxyeicosatetraenoicacid (12-HETE), and nitric oxide (NO) levels by UV irradiation of skin [7,8,9,10,11,12,13]. Prostaglandins (PGE_2_) are generated from arachidonate by the action of cyclooxygenase (COX-2) isoenzymes, which appears to be the dominant source of PGE_2_ formation in UV-induced erythema, i.e., sunburn or inflammation. Green tea catechins are selective for inhibition of COX-2 and could block their biosynthesis by anti-inflammatory actions in modulating the inflammatory response [14].

The efficacy of topically applied melatonin (N-acetyl-5-methoxytryptamine; a potent free radical scavenger) in the suppression of UV-induced erythema is evidently reported [15]. Additionally, oral and topical use of nicotinamide (vitamin B3 complex, a primary precursor of NAD) are reported to be effective in preventing UV-induced immunosuppression in human, however, dietary bioactive molecules such as green tea polyphenols, which possess anti-inflammatory, antioxidant, and immunomodulatory characteristics, are promising compounds due to the ability to attenuate the adverse effect of overexposure to ultraviolet radiation [16,17,18]. Although topical sunscreen use is still the first approach to preventing ultraviolet radiation damage to the skin, oral supplementation is an emerging candidate for skin protection. The current use of topical sunscreen for ultraviolet radiation protection has limitations because it is only effective for a finite period of time, can wear off, and requires frequent application. Antioxidant supplementation is recommended to provide a photo-protective effect against the reactive oxygen species formation upon ultraviolet radiation that initiates the detrimental signaling events in vasodilatation.

**Figure 1:**
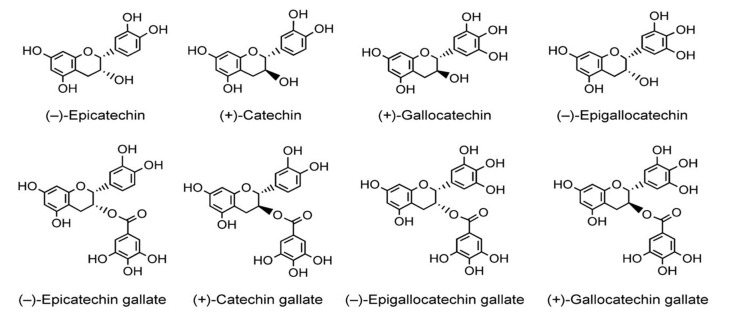
Chemical structure of different green tea catechins.

Green tea extracts derived from *Camellia sinensis* species of the Theaceae family have become a popular dietary source globally and are known for beneficial effects in several human diseases [19]. Most of the green tea catechins are monomeric flavanols called catechins. The four major catechin (see Figure 1) compounds are (−)-epigallocatechin-3-gallate (EGCG), (−)-epigallocatechin (EGC), (−)-epicatechin-3-gallate (ECG), and (−)-epicatechin (EC). EGCG is the most abundant and extensively studied catechin with potential therapeutic effects in skin diseases, immunosuppression, and carcinogenesis [19]. Several in-vitro and in-vivo animal and human studies demonstrated green tea catechin’s anti-inflammatory, antioxidant, photo-protective, and chemo-preventative effects after topical application and/or oral ingestion [20,21,22,23,24,25,26,27,28,29,30,31]. In a systematic review and meta-analysis, Tzellos et al. presented the efficacy, safety, and tolerability of green tea catechins in the treatment of external anogenital warts generally known as non-malignant skin tumor [32], while Scheinfeld [33] and Puviani et al. [34] described sinecatechin as a treatment of genital warts and external anogenital warts, respectively. Studies also observed regular intake of EGCG enriched green tea catechins increased the minimal dose of radiation required to induce erythema, suggesting green tea catechins can strengthen the skin’s tolerance by inhibiting the UV-induced skin damage [35,36,37]. Türkoğlu et al. [38] found that dermal gels of both black and tea extracts protected the skin against the harmful effects of UV radiation, such as erythema and premature aging of the skin. Similarly, Heinrich et al. [39] reported that green tea polyphenols provide photoprotection and improve physiological parameters of human skin. The available evidence regarding the protective effect of green tea catechin supplementation on the ultraviolet radiation damage to the skin reported a wider range of pro-inflammatory mediators involved in the sunburn response. However, there is a gap in knowledge regarding the impact of oral supplementation with green tea catechins on ultraviolet radiation-induced skin inflammation in human subjects. Therefore, we performed a systematic review and meta-analysis of selected studies to summarize the effect of oral green tea catechin supplementation on ultraviolet radiation-induced erythema.

## 2. Results

### 2.1. Literature Search Results

**Figure 2:**
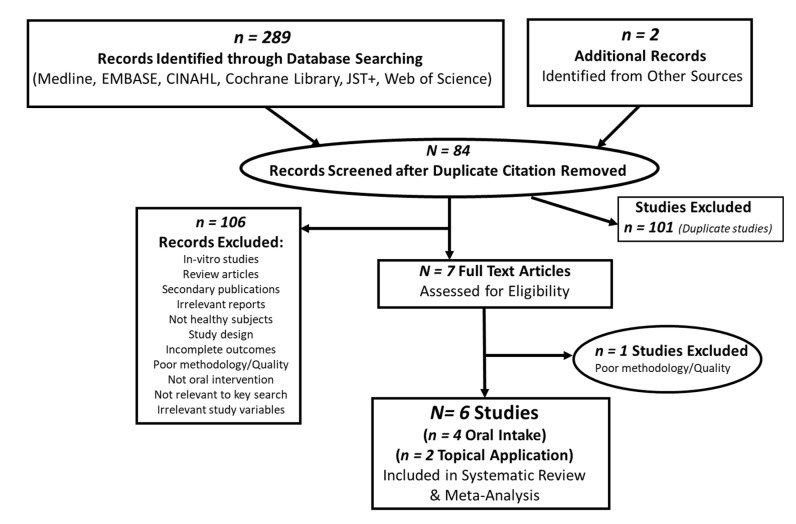
Schematic PRISMA flow diagram of included and excluded studies retrieved and identified by individual searches, and evaluated in the systematic review and meta-analysis of the effect of green tea catechins on the ultraviolet irradiation-induced erythema response.

The initial search yielded 289 citation records, and additional two records were identified from other sources as demonstrated in the flow of literature of retrieved studies (Figure 2). A total of 84 published records were screened after duplicate (*n* = 101), and non-relevant citations (*n* = 106) were removed. Seven full-text articles published in the peer-reviewed journals that examined the protective effect of green tea catechin intake on ultraviolet radiation-induced erythema outcomes were identified and reviewed. Further, one study was excluded after careful review based on the poor methodology and irrelevant information leaving data from six clinical studies for extraction. Further, available review articles and primary research citations were also searched manually, but no new suitable references were found. Of the six studies included in this systematic review and meta-analysis, a total of four clinical studies were identified as oral intakes, while two studies were the topical application of green tea catechins as detailed in the PRISMA flow chart (Figure 2). Relevant information from in-vitro studies and non-clinical animal and human clinical studies were systematically used to describe the effect of green tea catechins on ultraviolet radiation-induced erythema-related response. 

The data include the first name of the author, year of publication, study design, the formulation used, participant information, dosages, study duration, main diagnoses, study outcome, and related explanatory remarks (Table 1). Table 2 addresses the bias domain for each selected study by recording the method used to concealment of allocation, sequence generation for randomization, blinding implemented (to participants, personal, outcome assessors, data analyst, and others), incomplete outcome data, evidence of selective outcome reporting, and an additional source of bias [40,41,42]. The related items were classified as adequate, yes, inadequate no, or unknown/unclear (Table 2). 

### 2.2. Description of Retrieved Studies

The methodological characteristic of the eligible studies included in this systematic review and meta-analysis are described in Table 1, and further thoroughly evaluated in subsequent sections. The four studies identified as oral intake RCTs [20,21,22,23] reported the effect of oral green tea catechins on the bioavailability and cutaneous uptake of green tea catechins in human skin. The preliminary assessment of the impact of the green tea catechin oral intervention on the ultraviolet radiation-induced erythema was reported. In an open-label pre-test/post-test oral intervention study, Rhodes et al. (2013) examined the cutaneous uptake of green tea catechins on sunburn over a range of pro-inflammatory ultraviolet radiation doses [21] and explored the underlying mechanism of protection through modulation of ultraviolet radiation-induced pro-inflammatory mediators such as eicosanoids prostaglandin E_2_ (PGE_2_) and 12-hydoxyeicosatetraenoicacid (12-HETE).

It was hypothesized that the oral green tea catechins are bioavailable in human skin and thorough upregulated cyclooxygenase (COX) and lipoxygenase (LOX) metabolites inhibition may reduce the production of ultraviolet radiation-induced aforementioned pro-inflammatory mediators, which in turn may reduce the ultraviolet radiation-induced erythema response. The effect of the green tea catechin intake was reported for 10 healthy human subjects before and after green tea catechin supplementation by exposing buttock skin to ultraviolet radiation and quantify the level of erythema (sunburn). The modest level of green tea catechin intake at a daily dose of 540 mg was supplemented for 12 consecutive weeks. Results indicate that levels of metabolites of green tea catechins increased in the skin fluids after green tea catechin intake. Additionally, erythema levels were reduced after the 12 weeks supplementation period (*p* = 0.037). The pro-inflammatory markers PGE_2_ and 12-HETE were both found to be increased by ultraviolet radiation exposure, but 12-HETE levels were significantly reduced by the green tea catechin intake. Meanwhile, no effect on the PGE_2_ was noticed.

In a randomized, double-blind, placebo-controlled study, Farrar et al. (2015) reported a non-significant reduction in the erythema response within-group after oral intakes of green tea catechins (1080 mg/d) studied for a series of 10 ultraviolet radiation doses for 12 weeks among healthy adults (*n* = 25) [20]. However, erythema levels were non-significantly reduced after the 12 weeks of green tea catechin supplementation period compared to placebo. A similar inference was reported for the eicosanoid PGE_2_ and 12-HETE response to ultraviolet radiation-induced inflammatory challenges. Additionally, Farrar et al. (2018) recently performed a double-blind, randomized, placebo-controlled study in healthy adults who received either 1080 mg green tea catechins (*n* = 25) or placebo maltodextrin (*n* = 25) daily for 12 weeks [23]. A varied ultraviolet radiation dose was applied to buttock skin pre-and post-supplementation, and immune-histochemical staining with cyclobutane pyrimidine dimers (CPD) positive cell quantification was performed in the skin samples collected 24 h after ultraviolet radiation exposure. Following the intervention periods, there was no significant difference between green tea catechins and placebo groups in some CPD-positive cells in ultraviolet radiation irradiated epidermis at 24 h. Additionally, a significant difference was not observed in within-groups respective to both baseline and post-supplementation. Further ultraviolet radiation dose–response and further time point similarly found no significant effect of green tea catechin supplement on CPD. However, in another double-blind, randomized, placebo-controlled study, 60 female subjects were supplemented per day for 12 weeks with green tea polyphenols beverage containing 1402 mg green tea catechins [22]. Results showed that ultraviolet radiation-induced erythema decreased significantly (*p* < 0.001) in the green tea catechin intervention group by 25% after the 12 weeks, indicating that green tea catechin consumption effectively protects skin against harmful ultraviolet radiation. A statistically significant difference (*p* < 0.05) in the ultraviolet radiation erythema between the green tea catechins and placebo groups for implied ultraviolet radiation doses was also observed after the 6 weeks as well as after the 12 weeks of green tea catechin supplementation.

Katiyar et al. (1999) suggested that green tea catechins, especially EGCG, may be useful as a topical agent for protection against ultraviolet radiation-induced erythema possibly due to the anti-inflammatory effect of green tea catechins [24]. They reported that topical pretreatment with EGCG before ultraviolet radiation exposure significantly inhibited the development of erythema in human skin. In addition to erythema, the inhibition of ultraviolet radiation-induced epidermal cyclooxygenase activity was also reported with topical application of EGCG before the ultraviolet radiation exposure, as evident by the reduced pro-inflammatory markers PGE_2_ metabolite formation. In another study, they reported [25] that varying doses of topical treatment of green tea polyphenols to human skin before exposure to ultraviolet radiation at varying severity levels prevent the formation of ultraviolet radiation-induced cyclobutane pyrimidine dimers (CPD) in both epidermis and dermis, while also inhibiting erythema.

### 2.3. Meta-Analysis Results

Out of four eligible studies of oral intake of green tea catechins, three were randomized, double-blind, placebo-controlled studies [20,22,23], and one was an open-label intervention pre-/post supplementation study [21]. Additionally, two studies [24,25] were evaluated in the meta-analysis identified as topical application of green tea catechins on human subjects for the protection against ultraviolet radiation-induced erythema responses and pro-inflammatory mediators and metabolites. Studies with multiple dosages of green tea catechins, duration periods, and varied minimal erythema dosages (MED) were assessed as a separate study with suitable identifier tags. Although the included studies with differing MED doses were performed on the same individuals at the same time, the intra-individual correlation, even if it exists, is not necessary to be accounted for due to the random/fixed model nature of performed meta-analysis. An excellent agreement with the un-weighted κ value of 0.93 could be observed between the independent reviewers for full-text eligibility for included studies. 

#### 2.3.1. Erythema Index (Pre-/Post-Interventions)

**Figure 3:**
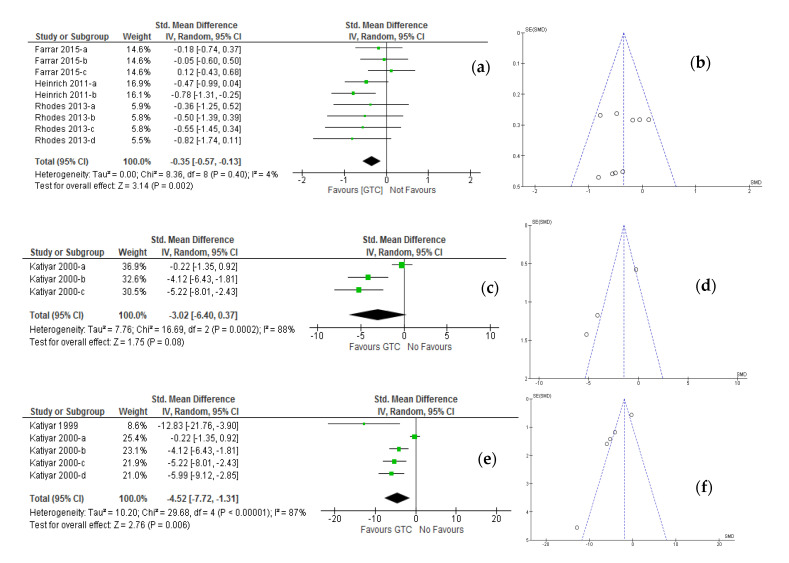
Meta-analysis of the effect of the varying dosages of green tea catechin supplementation on ultraviolet radiation-induced erythema response. (**a**) Forest plot of randomized controlled studies in healthy adults comparing the effect of before (pre-) and after (post-) oral green tea catechin interventions at MED challenge ranges between 1.0–2.5; (**b**) Funnel plot of representative oral green tea catechin supplementation studies (**c**) Forest plot of studies in healthy adults comparing the effect of before (pre-) and after (post-) topically applied green tea catechins at MED challenge ranges between 1.0–2.5; (**d**) Funnel plot of representative topically applied green tea catechin studies. (**e**) Forest plot of studies in healthy adults comparing the effect of before (pre-) and after (post-) topically applied green tea catechins at MED challenge ranges between 0.5–4.0; (**f**) Funnel plot of representative topically applied green tea catechin studies. ***Keys:*** Data are presented as mean and SDs, and the effect of studies is presented as weight (percentage) and standardized mean differences (SMDs) at 95% confidence intervals (CIs) using the generic inverse variance (IV) random-effects model. Inter-study heterogeneity was quantified by I^2^ statistics with significance *p* ≤ 0.10. The diamond represents the pooled effect estimated for the overall analysis.

The pre-test/post-treatment test design from three studies [20,21,22] of the oral intervention of green tea catechins, as well as a pre-test/post-treatment test design from two studies [24,25] of the topically applied green tea catechins are evaluated for their photo-protective effects against ultraviolet radiation-induced erythema as the primary outcome of the meta-analysis. The enrolled studies reported sufficient data along with adequate randomization and allocation concealment techniques, to allow for statistical pooling of pre-and post-intervention or application changes in the erythema index. A forest plot (Figure 3a) from three studies (MED range; 1.0–2.5) of oral intake studies with healthy participants (*n* = 175) revealed a highly significant pooling difference (*p* = 0.002) in pre-and post-intervention changes in erythema index (SMD: −0.35; 95% CI, −0.57 to −0.13; I^2^ = 4%, *p* = 0.40) in the random-effects model. On the other hand, visual inspection of the funnel plot (Figure 3b) reveals that data are systematically distributed around the middle and lower half of the funnel plot as the standardized mean difference for all studies, thus also indicates the absence of any publication bias. Similar significant pooling difference effect (*p* = 0.001) was noticed when fixed effects model was applied (SMD: −0.35; 95% CI, –0.56 to −0.14; I^2^ = 4%, *p* = 0.40). Additionally, the significant pooling difference could be observed when the mean difference (MD) was evaluated and compared for both random and fixed effects models in the meta-analysis (see Table 3). All studies included in this systematic review and meta-analysis measured the erythema index using solar simulator techniques (20–22), wherein the blue-light (mimicking sunlight) solar simulator was used to irradiate the skin, and skin color was evaluated by chromameter before and 24 h after irradiation at baseline and post-supplementation of green tea catechins.

The forest plot (Figure 3c) displaying an effect of topical green tea catechin application on erythema index revealed that pooled analysis of the two studies (MED range; 1.0–2.5) with 18 participants showed a non-significant pooling difference effect (*p* = 0.08) of green tea catechins on the reduction of ultraviolet radiation-induced erythema (SMD: −3.02; 95% CI, −6.40 to −0.37) in the random effect model. Substantial evidence of inter-study heterogeneity was present in the overall analysis (I^2^ = 88%, *p* ≤ 0.0002), which is also confirmed in the funnel plot (Figure 3d), which illustrates the heterogeneous nature of the data. The aforementioned results indicate that oral intervention of green tea catechins effectively inhibits the ultraviolet radiation-induced erythema with significance even at low-dose MED challenge, while topical application of green tea catechins inhibits the ultraviolet radiation-induced erythema, but no pooling significance could be reached at low-dose MED challenge levels. Furthermore, a significant pooling effect with topical application of green tea catechins on inhibition of ultraviolet radiation-induced erythema could only be observed in a meta-analysis of included studies with high-dose MED challenge levels (MED range; 0.5–4.0). The forest plot (Figure 3e) exhibiting an effect of topical green tea catechin application on erythema index revealed that pooled analysis (MED range; 0.5–4.0) from two studies with 28 participants showed a significant pooling difference effect (*p* =0.006) of green tea catechins on the reduction of ultraviolet radiation-induced erythema (SMD: −4.52; 95% CI, −7.72 to −1.31) in the random effect model. Again, an inter-study heterogeneity present in the overall analysis (I^2^ = 87%, *p* ≤ 0.00001), confirmed in the funnel plot (Figure 3f), illustrates the heterogeneous nature of the studies. 

**Figure 4:**
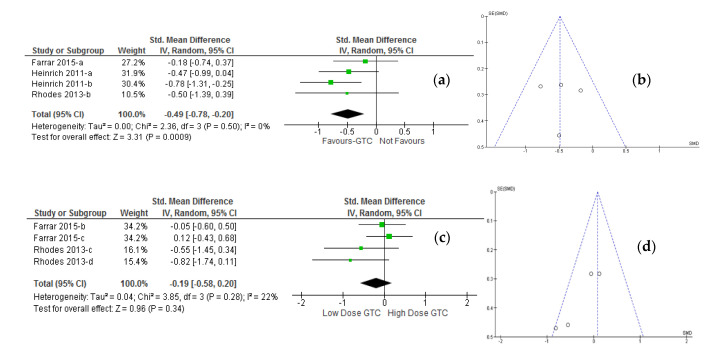
Meta-analysis of the effect of the oral green tea catechin supplementation on ultraviolet radiation-induced erythema response at varying UV-irradiation challenges. (**a**) Forest plot of randomized controlled studies in healthy adults comparing the effect of before (pre−) and after (post-) oral green tea catechin interventions at low-intensity MED challenge ranges between 1.25–1.30; (**b**) Funnel plot of representative oral green tea catechin supplementation studies. (**c**) Forest plot of randomized controlled studies in healthy adults comparing the effect of before (pre−) and after (post-) oral green tea catechin interventions at high-intensity MED challenge ≥1.50; (**d**) Funnel plot of representative oral green tea catechin supplementation studies. ***Keys:*** As illustrated in Figure 3.

Further, subgroup analysis was performed based on the study design. Categorical forest plot of meta-analysis from three studies with 95 participants (Figure 4a) revealed a highly significant pooled analysis difference of erythema index (*p* = 0.0009) with pre-and post-oral supplementation of green tea catechin at the MED between 1.25 and 1.30 times of ultraviolet radiation exposure of skin after 24 h of ultraviolet radiation challenge (SMD: −0.49; 95% CI, −0.78 to −0.20; I^2^ = 0%, *p* = 0.50) in both random and fixed effect models (see Table 3). On the contrary, the pooled analysis difference of erythema index quantified from pre-and post-intervention of green tea catechins after 24 h of ultraviolet radiation exposure of irradiated site of skin with 1.5 times or above MED of ultraviolet radiation for two studies with 70 participants (Figure 4c) showed no significant (*p* = 0.34) effect modification by higher MED of ultraviolet radiation exposure (SMD: −0.19; 95% CI, −0.58 to 0.20) in both random and fixed effect models, wherein studied MED was non-linearly associated with residual heterogeneity (I^2^ = 22%, *p* = 0.28). The related data evaluated as the pooled difference in mean difference (MD) with different models applied can be viewed in Table 3. Visual inspection of funnel plots (Figure 4b,d) suggests no asymmetry in both cases of erythema index evaluation when low or high MED challenges of ultraviolet radiation were respectively applied. However, the results confirmed that both low- and high dosages of green tea catechin oral supplementation are effective at low-dose ultraviolet radiation irradiation-induced erythema. Furthermore, the low dose of green tea catechin oral supplementation was found effective at a high intensity of ultraviolet radiation irradiation-induced erythema threshold, and high doses of green tea catechin oral supplementation were found with the least suppressive effect at high-intensity ultraviolet radiation-induced erythema level.

#### 2.3.2. Erythema Index (Green Tea Catechins vs. Placebo)

**Figure 5:**
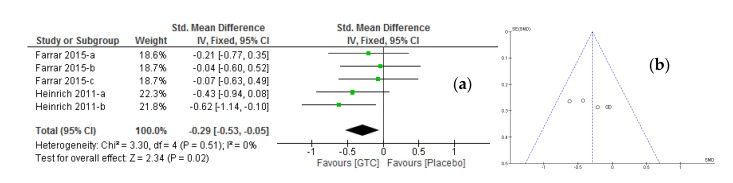
(**a**) Forest plot of randomized controlled studies in healthy adults comparing the effect of oral green tea catechin interventions with placebo showing effectiveness at low intensity (MED challenge ranges between 1.25–1.30) UV-irradiation-induced erythema compared to placebo; (**b**) Funnel plot of representative oral green tea catechins and placebo supplementation studies. ***Keys***: As illustrated in Figure 3.

A meta-analysis of two randomized, double-blind, and placebo-controlled studies [20,22] was pooled to evaluate the effect of green tea catechin intervention on the ultraviolet radiation-induced erythema index (Green tea catechins, *n* = 135; Placebo, *n* = 132). The forest plot (Figure 5a) revealed a significant pooling difference (*p* = 0.02) in erythema index reduction in favor of green tea catechins compared to placebo in both the random and fixed effect models (SMD: −0.29; 95% CI, −0.53 to −0.05; I^2^ = 0%, *p* = 0.51). The related summarized data are listed in Table 3. No heterogeneity was observed and the funnel plot shown in Figure 5b further illustrates the non-heterogonous nature of the data as no asymmetry in the erythema index was noticed. Both Egger’s and Begg’s tests indicated no publication bias (*p* = 0.23 and *p* = 0.34, respectively). The results confirm that green tea catechin oral supplementation are effective at low-intensity ultraviolet radiation-induced erythema (MED range; 1.25–1.30) compared to placebo.

#### 2.3.3. Pro-Inflammatory Mediators (Pre-/Post-Oral Green Tea Catechin Interventions)

**Figure 6:**
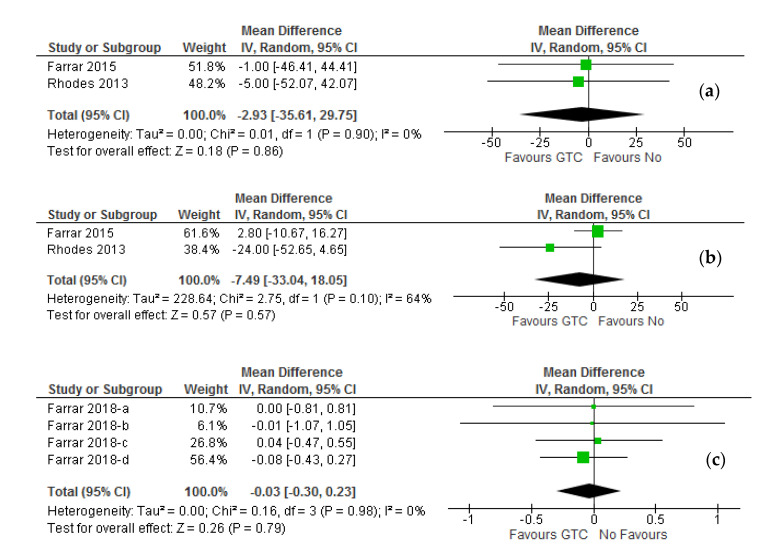
Forest plot of randomized controlled studies in healthy adults comparing the effect of before (pre−) and after (post-) oral green tea catechin interventions at the varying intensity of MED challenge showed a nonsignificant inhibition of ultraviolet radiation-induced pro-inflammatory mediators. (**a**) PGE_2_; (**b**) 12-HETE, and (**c**) CPDs. ***Keys:*** As illustrated in Figure 3. The effect of studies is presented mean differences (MDs) at 95% confidence intervals (CIs) using the generic inverse variance (IV) random-effects model.

The data retrieved from two pre-/post-green tea catechin intervention pilot studies (30 participants) [20,21] was pooled to quantify the effect of green tea catechin incorporation into the skin for modest reduction of an active vasodilator prostaglandin E_2_ (PGE_2_). The forest plot (Figure 6a) revealed a non-significant reduction (*p* = 0.86) in pooling difference (30 participants) in PGE_2_ concentration in favor of green tea catechin intervention in both the random and fixed effect models (MD: −2.93 pg/μL; 95% CI, −35.61 to 29.75; I^2^ = 0%, *p* = 0.91). Similarly, 12-hydroxyeicosatetraenicacid (12 HETE), a potent leukocyte chemoattractant in the sunburn response, was pooled to evaluate the data retrieved from two selected pre-/post-green tea catechin intervention studies with 34 participants.

Although a reduction in the pooling difference in 12HETE concentration could be observed in favor of green tea catechin intervention (Figure 6b) in both the random and fixed effect models (MD: −7.49 pg/μL; 95% CI, −33.04 to 18.05), a significant pooling difference was not reached (*p* = 0.57). However, there was substantial heterogeneity with significance in the pooled analysis (I^2^ = 64%, *p* =0.10) indicating that a single study exerted influence on the overall meta-analysis result. On the other hand, a pooling of 35 participants in the meta-analysis [23] showed that an oral green tea catechin intake prevents the formation of ultraviolet radiation-induced cyclobutane pyrimidine dimers (CPD). 

The forest plot (Figure 6c) revealed a non-significant reduction in the number of CPD positive cells formation (*p* = 0.79) in favor of green tea catechin intake in both random and fixed effect models (MD: −0.03; 95% CI, −0.30 to 0.23; I^2^ = 0%, *p* = 0.98). Thus, no effect of green tea catechin supplement could be observed on the reduction of some CPD formation in the epidermis of irradiated skin with moderate ultraviolet radiation doses and irradiation time of duration. The related detailed meta-analysis is compiled in Table 4.

## 3. Materials and Methods

The present systematic review and meta-analysis are conducted according to the guidelines of the PRISMA statement as well as the guidelines of the Cochrane Handbook of Systematic Reviews and Intervention [40,43,44].

### 3.1. Literature Search Strategy

A comprehensive literature search was initially carried out in November 2020 to identify the relevant observational studies using suitable identical keywords for all the search engines, and reconfirmed in April 2021 for any updates. The electronic databases Medline (PubMed; National Library of Medicine database), Cochrane Library of a central register of controlled studies, CINAHL database, EMBASE Classic database network, and Web of Science database were searched for English language publications. Meanwhile, JST (JICST, JSTPLUS, and JMEDPLUS) databases were searched using identical keywords for retrieval of the Japanese literature, with no restriction of date of publication. Specifically, the search text terms include melanin OR (skin) OR (Beauty) OR (whitening) OR (erythema) OR (MED) OR (skin burn) OR (tanning) OR (sunburn) OR (DNA damage), and were combined using the set operator AND with studies identified with the following terms: (Camellia Sinensis) OR (catechin) OR (green tea) OR (tea polyphenols) OR (green tea extract) OR (green tea catechin) OR (GTE) OR (GTC) OR (EGCG) OR (epigallocatechin gallate) OR (epigallocatechin 3-gallate) OR (epigallocatechin-3-gallate) OR (epigallocatechin-3-o-gallate) OR (epigallocatechin gallate) OR (galloylepigallocatechin). The search was restricted to human clinical studies. Further, a multistep search approach was considered to retrieve relevant studies through additional manual searching the references from retrieved clinical studies, and review articles, theses, and dissertations or abstract books of conference proceedings to further identify possible additional potentially eligible studies. The search results were supplemented with searches in Google Scholar for a recursive search of the literature using the bibliographies of all identified relevant studies. 

### 3.2. Study Selection Criteria, Data Extraction and Assessment of the Risk of Bias and Outcome

In agreement with the quality reporting of meta-analysis, well-defined inclusion and exclusion criteria were employed for the selection of the studies. Two researchers carried out independent screening and quality assessment according to the guidelines of PRISMA. Limitations were set up for original articles and short communications, and therein only published studies were considered whilst the preliminary drafts were excluded. The included studies were required to be either: (i) Clinical studies on human subjects only, or (ii) be a study that investigated the association between the intake of green tea catechins and protection against ultraviolet radiation-related parameters, i.e., erythema index or minimal erythema dose, or (iii) be a randomized study or controlled intervention study, or (iv) parallel or crossover or pre-test/post-test design study, or (v) be a study of any duration with either single or chronic doses of green tea catechins, or (vi) be a direct comparison of green tea catechins vs. placebo or high vs. low dosages of green tea catechins, or (vii) be a study with quality greater than or (equal to 3 points judged by Jadad score [45]), or (viii) be a study with reasonably tolerable dosages of green tea catechins either as a capsule, regular beverage, or tablet, or (ix) be an ultraviolet radiation-related study ethically approved by the authorized institutions. The primary outcomes assessed were the effect of green tea catechin protection against ultraviolet radiation-related parameters erythema index based on minimal erythema dose compared before and after intake of green tea catechins, as well as at varying ultraviolet radiation levels to quantify the effectiveness of green tea catechins in the ultraviolet radiation-induced erythema. Outcomes were also assessed while the effect of green tea catechins compared with placebo on protection against ultraviolet radiation-induced erythema. Additionally, the secondary outcomes concerning the DNA damage usually associated with ultraviolet radiation-induced erythema are separately presented to justify the erythema outcomes in this systematic review and meta-analysis.

To eliminate the possibility of variation usually caused by systematic errors in study approach patterns and the execution, the two independent researchers assessed the quality of the retrieved articles according to pre-specified selection criteria, and final data were extracted as mean (M) along with a standard deviation (S.D). Arbitration was sought within authors to resolve any specific issues and discrepancies with pointed discussions. Further, an un-weighted κ calculation was performed to quantify the agreement between the two reviewers, wherein the κ values above >0.80 were considered to reflect an acceptable agreement. Full texts of the selected studies were doubly checked for their suitability for presented systematic review and meta-analysis. Additionally, the pertinent clinical data regarding general background information concerning the studies were extracted. The risk of publication bias was assessed according to the standards of the Cochrane handbook guidelines [41]. Finally, the funnel plots with forest plots were assessed for evidence to verify the publication bias [46]. Additionally, the Jaded score [45] was also ascribed to assess the quality of the included studies, wherein studies accumulated 1 point for each area addressed in the study design, preferably randomization, blinding, reporting of withdrawals allocation of concealment and random numbers generation, with a possible score of between 0–5 depending on the quality of the study, wherein the higher number represented a better quality of the included study [47].

### 3.3. Data Management and Statistical Procedures

The treatment effect of green tea catechins on the defined primary outcome was calculated as the difference between the end of intervention values for the intervention and placebo groups. The net changes in each of the study parameters as means (M) and standard deviation (SDs) were used to calculate the pooled effects. The variance was calculated from the SD and standard errors (SE) of end of intervention values or the 95% confidence intervals (95% CIs) when these values were not available. In case the data were reported as median and IQR, it converted to mean and SD. When SDs were not directly available, their values were calculated from SE, 95% CIs, or *p* values if necessary. In crossover studies, the mean and SD, SE, or CI of intervention and control periods were extracted and analyzed in a separate meta-analysis. The percent changes in mean and SD values were entirely excluded for analysis, and when the end of intervention endpoint data was not available, the results were described narrative only.

The pre-specified exploratory outcome data were analyzed for meta-analysis using Review Manager Software (RevMan, version 5.4.1; http:/ims.cochrane.org/revman; accessed on 1 April 2020) provided by Cochrane collaboration, London, United Kingdom. The statistical analysis was performed following the guidelines in the Cochrane Handbook for systematic reviews of intervention and respective meta-analyses. Weighted mean differences (MDs) with corresponding 95% CIs were calculated for continuous/dichotomous outcomes of net changes in the effective values for continuous data [48,49,50,51]. When outcome data were reported in different units to measure the same size effect or studies reported with sufficient changes, the pooled effects were calculated using the weighted standardized mean difference (SMDs) with corresponding 95% CIs [41,48]. Therefore, SMD expresses the size of the intervention effect in each study relative to the variability observed in that study [52].

Inconsistencies between studies were assessed with the use of the I^2^ statistics, which measures the extent of variability across studies examined for the heterogeneity between randomized controlled studies. Significant heterogeneity was defined as I^2^ > 50%. The χ^2^ test with *p*-values of <0.10 and values of I^2^ > 50% indicated significant heterogeneity according to the Cochrane handbook of systematic reviews [48,49]. A fixed-effect model was used to check for robustness and potential outliners of a pooled data estimate of the MD and SMD, whereas in the case of significant heterogeneity, results were further confirmed by using a random effect model [41]. Thus, to display clarity of the information on data analysis, we provided the results calculated from both fixed-effect and random effect models to quantify the effectiveness of green tea catechins in the ultraviolet radiation-induced erythema. Meta-analysis was performed using the continuous effect model, and data could be appropriately pooled for subgroup analyses. A two-tailed *p* < 0.05 was considered statistically significant. Heterogeneity was further explored by using the dosage effect correlation to verify the relationship between dosages of green tea catechins as well as with identical minimal erythema dose to summarize the ultraviolet radiation-induced erythema outcomes. The selected articles were categorized into oral intakes and topical applications, respectively, based on their relevance to defined inclusion/exclusion criteria.

## 4. Discussion

Sunburn is an erythema reaction known to be induced by increased eicosanoids prostaglandin E_2_ (PGE_2_), and nitric oxide levels in the skin exposed to ultraviolet radiation [8,9,10,11,12,13,53]. Ultraviolet radiation stimulates epidermal keratinocytes to exhibit mRNA of cyclooxygenase 2 (COX-2) and inducible nitric oxide synthase (iNOS), which produce PGE_2_ and NO, respectively. Additionally, the repeated sub-erythema ultraviolet radiation exposures on human skin have been shown to induce significant DNA damage in epidermal cells. Further, the deleterious effects of the release of NO have been implicated in tissue damage and inflammation. Green tea catechins are the prominently consumed botanical in dermatology, especially against acne-causing bacteria because it neutralizes the free radicals, reduces inflammation, and slows the deterioration of the skin conditions. Earlier research found the antioxidant capacity of green tea provided direct scavenging activity against NO in-vitro [54] and EGCG inhibited ultraviolet-B-induced activation of NFκB, sequentially blocking inducible iNOS preventing the production of NO [55,56]. Although green tea catechins reduce ultraviolet radiation-induced inflammation in experimental animal models and have been successfully authenticated particularly in-vitro, introduced in cosmetology as an anti-aging active ingredient for topical use, there is much less information about the efficacy of oral treatment for photo-protection in human in-vivo studies. Whether topically applied or orally consumed, green tea catechins have emerged as a natural and multidimensional powerhouse shown to exhibit significant antioxidant, chemo-preventative and anti-inflammatory activity, and affecting cell proliferation pathways.

We performed the systematic review followed by analysis and summarized the available published literature examining the efficacy of oral green tea catechin intervention on the inhibition of ultraviolet radiation-induced erythema (sunburn). To our knowledge, this is the first meta-analysis to assess the effectiveness of green tea catechins specifically on measures of ultraviolet radiation-induced erythema and related pro-inflammatory mediators.

The green tea catechin intake contributed to protecting skin against harmful ultraviolet radiation and resulted in the incorporation of catechin metabolites into human skin associated with abrogated ultraviolet radiation-induced PGE_2_, 12 HETE, and CPD that may contribute to protection against sunburn inflammation and potentially longer-term ultraviolet radiation mediated damage. The levels of 12 HETE markers of inflammation were reduced by the green tea catechin supplement, however, limited effects on the PGE_2_ and CPD were observed. The reduction of such ultraviolet radiation-induced pro-inflammatory mediators is important because ultraviolet radiation exposure-induced injury can initiate oxidative stress-stimulated expression followed by upregulation of cyclooxygenase and/or lipoxygenase activities. This may lead to the increased production of PGE_2_ and 12 HETE, which may function as a mitogen, inhibit apoptosis, and may enhance the DNA adduct formation via inflammation in the sunburn response. In terms of pro-inflammatory eicosanoids, much focus has been on the involvement of PGE_2_ in ultraviolet radiation-mediated erythema. However, COX-2 inhibitors only partially suppress ultraviolet radiation-induced erythema [8,57]. Another possibility that 12-HETE could be involved in the later erythema, and thus suppression of the LOX-derived mediator 12-HETE may operate an anti-inflammatory effect reflected in the reduced erythema, including a potential effect through reduced leukocyte attraction. Additionally, the reduction of CPD common ultraviolet products is essential, since the DNA is a chromophore for ultraviolet radiation. Such a direct interaction produces cyclobutane pyrimidine dimers, mutagenic photoproducts that may lead to tumor initiation, immune suppression, interference with base pairing during DNA replication, leading to mutation and skin cancer promotion. 

Earlier findings have also demonstrated that EGCG has the potential to inhibit UVB-mediated activation of cellular signaling pathways in human keratinocytes, and which are deleterious for the normal skin cells [58,59]. The treatment of normal human epidermal keratinocytes (NHEK) with EGCG reduced UVB-mediated phosphorylation of mitogen-activated protein kinases (MAPKs) and activation nuclear factor kappa B (NF-κB) signaling pathway. The regular intake of green tea polyphenols as protective agents against the adverse effects of UV radiation might be helpful for the prevention of any dysregulation of cellular signaling pathways that result in photocarcinogenesis in humans. Thus, all mechanistic studies included in this systematic review and meta-analysis to examine the impact of oral green tea catechin supplementation on the cyclooxygenase and lipoxygenase metabolites fairly contributed to the outcomes that green tea catechins indeed modestly protect against ultraviolet radiation-induced erythema. This evidences a measured reduction in erythema index at a higher dose of MEDs even at a low dose of 540 mg green tea catechins per day supplementation. It is also important to acknowledge that there could be the possibility of a nonlinear dose–response effect in the studies with a higher dose of green tea catechin supplementation because a higher concentration of nutritional supplements is sometimes reported to be less effective. Studies also included in this meta-analysis reported a suppressive effect of ultraviolet radiation-induced erythema threshold at low-dose MED challenge with the use of a higher dose of green tea catechins (1402 mg/day and 1080 mg/day, having 980 mg and 436 mg EGCG, respectively).

Oral intake of green tea catechins significantly prevented ultraviolet radiation-induced depletion of antioxidant enzymes, but its photo-protective efficacy was lesser than that of topical applications of green tea catechins due to lower bioavailability in skin target cells. In this view, inter-individual variability in the type of catechins and their metabolites in skin post-supplementation may effectively influence the outcomes. Clarke et al. (2016) evaluate the oral bioavailability of green tea catechin metabolites in the skin by assessing skin biopsies and blister fluids (pre- and post-ultraviolet radiation exposure) and compared green tea catechin metabolites in plasma and urine to understand the novel link between catechin metabolites derived from the skin and gut microbiota [60]. The results of the study suggest that green tea catechin metabolites can reach the skin post-supplementation in the unconjugated (free) form and as conjugated derivatives, with a clear overlap between the metabolites present in the plasma samples and the blister fluid. Thus, any potential lowering of the ultraviolet radiation-induced inflammatory markers or improvement in the erythema index reported as outcomes in this meta-analysis following the varied dose of green tea catechin oral supplementation is well explained. It could be considered that the gastrointestinal environment and epithelial transport process might affect the molecular modification and biological activity of orally ingested green tea catechins. Possibly, some of the green tea catechin components could not be transported across the skin tissue cells and metabolized during passage through the cells. However, it is important to emphasize that oral green tea catechins could be an adjunct for topical sunscreen even though the topical application of green tea catechins potentially has higher local skin concentrations of green tea catechins that are considered to be protected from the ultraviolet radiation-induced immunosuppressive state. Low-dose vitamin C was also ingested along with green tea catechins in the studies included in this meta-analysis, wherein the low dose of vitamin C is reported to stabilize the green tea extract in the gut lumen but has no impact itself on ultraviolet radiation erythema [61,62]. Additionally, in another report, vitamin C has been shown to provide a stabilization effect, increasing catechin bioavailability through enhancing accessibility and intestinal uptake [63]. The instability of green tea catechins is normally induced by the degradation and oxidation of tea catechins. Conversely, although it cannot be completely excluded, the combined use of green tea catechins and vitamin C may, in turn, provide greater photoprotection than green tea catechins alone. Further, it is worth mentioning that the white Caucasian males and females subjects of sun-reactive skin type I (easy sunburn, minimal tanning) and type II (burns and peels often, and rarely tans) according to Fitzpatrick [64], who took part in the studies included in this meta-analysis with the consumption of green tea catechins [20,21,22] to evaluate the effect of green tea catechins on erythema response. It is reported that several variables related to the function and structure of skin such as a decrease in viscoelasticity and biological elasticity, reduction in skin roughness, increased skin density, and skin hydration were modulated by the green tea catechin intervention [22].

The present meta-analysis includes relatively narrow search criteria of green tea catechin effects on erythema dose–response. In the analysis, we employed a reproducible search of all existing evidence from the literature to maximize the generalizability of the outcomes. Although the inconsistency of the estimates across studies resulted in a varied heterogeneity for few outcomes, the subgroup analyses could explain the observed substantial heterogeneity. Overall, the certainty of the available evidence was evaluated moderate for erythema dose–response outcome. Notably, although the magnitude of the effect is modest and sometimes lacks clinical significance on an individual level, our meta-analysis indicates the significant pooling effect of included studies for the outcomes. The meta-analysis results also revealed, even at low-dose MED challenge, the oral green tea catechin intervention significantly inhibits the ultraviolet radiation-induced erythema, whereas topically applied green tea catechins inhibit the ultraviolet radiation-induced erythema. However, no pooling significances could be reached at low-dose MED challenge levels. Conversely, a significant pooling effect with topically applied green tea catechins on inhibition of ultraviolet radiation-induced erythema could be observed in a meta-analysis of included studies with high-dose MED challenges (MED range; 0.5–4.0). Our meta-analysis confirmed the effectiveness of both low and high doses of oral green tea catechins for low-dose ultraviolet radiation-induced erythema response. However, the low dose of green tea catechin oral supplementation was moderately effective at a high intensity of ultraviolet radiation-induced erythema threshold. In contrast, high doses of green tea catechin oral supplementation were found to have the least suppressive effect on high-intensity ultraviolet radiation-induced erythema response. Notably, the meta-analysis results confirm that the oral intake of green tea catechins is highly effective in low-intensity ultraviolet radiation-induced erythema response (MED range; 1.25–1.30) compared to placebo. Thus, the overall outcomes indicate that green tea catechin oral supplementation modestly yet significantly protects ultraviolet radiation-induced erythema response in humans.

## 5. Conclusions

There has been limited research on the effect of oral supplementation with green tea catechins on ultraviolet radiation-induced skin inflammation in human subjects. The limitations of topically applied sunscreens are that they offer inadequate protection against the detrimental effects of ultraviolet radiation on cutaneous immunity, which has given rise to research to develop systemic sun protection. It is thought that systemic photo-protection may provide a constant low level of protection to the entire body surface. Our meta-analysis findings also suggest that the green tea catechins have the potential to be a preventative measure against ultraviolet radiation-induced skin damage in humans and continuous low-level sun protection through dietary green tea catechins might act as an effective adjunct or suitable alternative to the more intermittently applied prominent topical sunscreens protection. The outcomes of this meta-analysis also revealed that regular intake of as low as 540 mg of green tea catechins per day could be beneficial for the protection against ultraviolet radiation-induced erythema, wherein green tea catechin metabolites are bioavailable at the dermis and epidermis levels of the skin, and thus increase the minimal dose of radiation (MED) required to induce erythema. This in turn suggests that green tea catechins can strengthen the skin’s tolerance to ultraviolet radiation-induced skin damage from radiation through the prevention of the ultraviolet radiation-induced perturbation of epidermal barrier functions.

## Figures and Tables

**Table 1:** Characteristics features and methodological assessments of the included studies evaluated in the meta-analysis of green tea catechin’s effect on ultraviolet irradiation-induced erythema response in healthy humans.

Study [Reference]	Study Design	Study Population	Dosage/ Day	Study Duration	Study Descriptions ^#^	x. MED	Meta-Analysis Parameters	Study Details/ Remarks
Farrar et al., 2015 [20]	Randomized Double-blind Placebo controlled (RCT)	Healthy adults (male and female) GTC 25; Placebo 25 Age 18–65 years	Capsules GTC, 1080 mg	12 weeks	Farrar 2015-a Farrar 2015-b Farrar 2015-c	1.25–1.30 1.70–1.75 2.0–2.3	Ultraviolet Radiation-induced Erythema index △E-value PGE_2_ 12-HETE	Studied 10 dosages of simulated Ultraviolet radiation (7–80 mJ/cm^2^); irradiated sites were examined after 24 h; skin biopsy, analysis of skin blister fluid, urinary analysis of GTC metabolites; Cutaneous production of eicosanoids, Ultraviolet radiation-induced erythema response (threshold value)
Rhodes et al., 2013 [21]	Open Oral Intervention Pre- and post- intervention	Healthy adults (male and female) GTC 16 Age 29–59 years	Capsules GTC, 540 mg	12 weeks	Rhodes 2013-a Rhodes 2013-b Rhodes 2013-c Rhodes 2013-d	1.0–1.1 1.25–1.30 1.60–1.65 2.0–2.1	Ultraviolet Radiation -induced Erythema index △E-value PGE_2_ 12-HETE	Studied 10 dosages of simulated Ultraviolet radiation (6.67–68 mJ/cm^2^); irradiated sites were examined visually after 24 h; Skin tissues and blister fluid sampling; evaluated cutaneous eicosanoid levels; Urinary metabolites; Ultraviolet radiation-induced erythema Index (threshold value)
Heinrich et al., 2011 [22]	Randomized Double-blind Placebo controlled (RCT)	Healthy adults (female) GTC 30; Placebo 30 Age 40–65 years	Capsules GTC, 1402 mg	6 weeks, and 12 weeks	Heinrich 2011-a Heinrich 2011-b	1.25 1.25	Ultraviolet Radiation -induced Erythema index △a-value	MED estimation; Irradiation with 1.25 MED at wk 0, wk 6, and wk 12 (time points); Erythema measured before and 24 h after exposure; measurement of reddening (a-value); skin elasticity, structure and texture, and hydration and transepidermal water loss.
Farrar et al., 2018 [23]	Randomized Double-blind Placebo controlled (RCT)	Healthy adults (male and female) GTC 20; Placebo 24 Age 18–65 years	Capsules GTC, 1080 mg	12 weeks	Farrar 2018-a Farrar 2018-b Farrar 2018-c Farrar 2018-d	3.0 2.0 2.0 2.0	CPD	Ultraviolet radiation-induced epidermis compared post-supplementation for Immunohistochemically staining with CPDs; Placebo vs. GTC; up to 3xMED erythema dose; studied photoprotection of skin from direct DNA damage.
Katiyar et al. 1999 [24]	In-vivo Topical application	Healthy adults (male and female) *n* = 4 Age 18–65 years	GTC (EGCG) 3 mg/2.5 cm^2^ skin area	Single-dose	Katiyar 1999	4.0	Ultraviolet Radiation -induced Erythema index △E-value PGE_2_	Topical application of GTC (EGCG, 3 mg/2.5 cm^2^ skin area); Ultraviolet radiation-induced erythema response; Exposures at 4.0 MED; studied MPO and leukocyte inhibition; Cyclooxygenase activity (prostaglandin metabolites)
Katiyar et al. 2000 [25]	In-vivo Topical application	Healthy adults (male and female) *n* = 6 Age 18–65 years	GTC (EGCG) 3 mg/2.5 cm^2^ skin area	Single-dose	Katiyar 2000-a Katiyar 2000-b Katiyar 2000-c Katiyar 2000-d	0.50 1.0 2.0 4.0	Ultraviolet Radiation -induced Erythema index △E-value CPD	Topical application of GTC (3 mg/2.5 cm^2^ skin area); Ultraviolet radiation-induced erythema response; Exposures at 0.5–4.0 MED; Skin punch biopsies; Immunostaining of CPDs (Ultraviolet radiation dose-dependent at epidermis level)

PGE_2_, Prostaglandin E_2_; 12-HETE, 12-Hydroxyeicosatetraenoicacid; CPD, Cyclobutane pyrimidine dimers; MED = Minimal Erythema Dose; GTC= Green Tea Catechins. ^#^ Study descriptions: Substudies assigned as a, b, c, d are the included data trial points of selected studies.

**Table 2:** Assessment of validity, risk of bias, and Jadad score of the included eligible studies evaluated in the meta-analysis.

Author and Year	Farrar et al., 2015	Rhodes et al., 2013	Heinrich et al., 2011	Farrar et al., 2018	Katiyar et al., 1999	Katiyar et al., 2000
Reference No.	[20]	[21]	[22]	[23]	[24]	[25]
Study type	Oral	Oral	Oral	Oral	Topical	Topical
Allocation Concealment	Adequate	Adequate	Adequate	Adequate	Adequate	Adequate
Sequence Generation	Yes	Yes	Yes	Yes	No	No
Randomization	Yes	Yes	Yes	Yes	No	No
Blinding	Yes	Yes	Yes	Yes	No	No
Incomplete Outcome	No	No	No	No	No	No
Withdrawal/Other reporting	Yes	Yes	Yes	Yes	Yes	Yes
Jadad Score	5	4	5	5	2	2

**Table 3:** A meta-analysis of included studies investigating the effect of green tea catechin supplementation on the ultraviolet irradiation-induced erythema response in humans ranked by selected study characteristics.

Study Characteristics	Outcomes Measured	Study/ Subjects	Meta-Analysis (Estimation)	Test of Heterogeneity			Pooled *p*-Value
			Inverse Variance, Net Change (95% CI)	Chi-Square Test	*p*	I^2^ (%)	
Oral Intake							
Pre-test/ GTC-test (Controlled test)	(i) GTC effect on Erythema Index at varied MED (Between 1.0–2.5)	9/175	^a^ SMD: −0.35 [−0.57, −0.13]	8.36	0.40	4	0.002
			^b^ SMD: −0.35 [−0.56, −0.14]	8.36	0.40	4	0.001
			^a^ MD: −1.01 [−1.56, −0.47]	7.60	0.47	0	0.003
			^b^ MD: −1.01 [−1.56, −0.47]	7.60	0.47	0	0.003
	(ii) GTC effect on Erythema Index at identical MED (1.25 ≤ 1.30)	4/95	^a^ SMD: −0.49 [−0.78, −0.20]	2.36	0.50	0	0.0009
			^b^ SMD: −0.49 [−0.78, −0.20]	2.36	0.50	0	0.0009
			^a^ MD: −0.99 [−1.54, −0.45]	1.93	0.59	0	0.0004
	(iii) Dose–response of GTC on Erythema Index at higher MED	4/70	^a^ SMD: −0.19 [−0.58, −0.20]	3.85	0.28	22	0.34
			^b^ SMD: −0.16 [−0.49, 0.18]	3.85	0.28	22	0.36
			^a^ MD: −5.24 [−15.16, 4.68]	4.49	0.21	33	0.3
			^b^ MD: −4.02 [−11.74, 3.71]	4.49	0.21	33	0.31
	(≥1.50)						
RCT studies (Placebo-Controlled)	(i) GTC effect on Erythema Index compared to Placebo at varied MED (Between 1.0–2.5)	5/P132 5/GTC 135	^a^ SMD: −0.29 [−0.53, −0.05]	3.30	0.51	0	0.02
			^b^ SMD: −0.29 [−0.53, −0.05]	3.30	0.51	0	0.02
			^a^ MD: −1.38 [−2.28, −0.47]	0.57	0.97	0	0.003
			^b^ MD: −1.38 [−2.28, −0.47]	0.57	0.97	0	0.003
Topical Application							
Pre-test/ GTC-test (Controlled test)	GTC effect on Erythema Index at varied MED (Between 0.5–4.0)	5/28	^a^ SMD: −4.52 [−7.72, −1.31]	29.68	<0.00001	87	0.006
			^b^ SMD: −1.98 [−2.89, −1.07]	29.70	<0.00001	87	<0.0001
			^a^ MD: -6.68 [−11.37, −1.99]	372.5	<0.00001	99	0.005
			^b^ MD: −4.38 [−4.84, −3.91]	0.57	<0.00001	99	<0.00001
	GTC effect on Erythema Index at MED ≤ 2.0	3/18	^a^ SMD: −3.02 [−6.40, 0.37]	16.70	0.0002	88	0.08
			^b^ SMD: −1.48 [−2.44, −10.52]	16.70	0.0002	88	0.002
			^a^ MD: −3.44 [−7.01, 0.13]	83.60	<0.00001	98	0.06
			^b^ MD: −2.27 [−2.79, −1.75]	83.60	<0.00001	98	<0.00001

^a^ Random Effect Model; ^b^ Fixed Effect Model; SMD: Standardized Mean Difference; MD: Mean Difference. GTC = Green Tea Catechins.

**Table 4:** Meta-analyses of ultraviolet radiation-induced pro-inflammatory mediator eicosanoids and pyrimidine dimers ranked by green tea catechin supplemented studies on healthy humans.

Study Characteristics	Outcomes Measured	Study/Subjects	Meta-Analysis (Estimation)	Test of Heterogeneity			Pooled *p*-Value
			Inverse Variance, Net Change (95% CI)	Chi-Square Test	*p*	I^2^ (%)	
Pre-test/ GTC-test (Controlled test)	(i) GTC effect on PGE_2_	2/30	^a^ MD: −2.93 [−35.61, 28.75]	0.01	0.9	0	0.86
			^b^ MD: −2.93 [−35.61, 28.75]	0.01	0.9	0	0.86
	(ii) GTC effect on 12-HETE	2/34	^a^ MD: −7.49 [−33.04, 18.05]	2.75	0.1	64	0.57
			^b^ MD: −2.05 [−14.24, 10.14]	2.75	0.1	64	0.74
	(iii) GTC effect on CPD	4/35	^a^ MD: −0.03 [−0.30, 0.23]	0.16	0.98	0	0.79
			^b^ MD: −0.03 [−0.30, 0.23]	0.16	0.98	0	0.79
RCT studies (Placebo-Controlled)	(i) GTC effect on PGE_2_ compared to Placebo	1/P21 1/GTC 20	^a^ MD: −12.80 [−37.08, 11.48]	na	na	na	0.3
			^b^ MD: −12.80 [−37.08, 11.48]	na	na	na	0.3

^a^ Random Effect Model; ^b^ Fixed Effect Model; SMD: Standardized Mean Difference; MD: Mean Difference; PGE_2_, Prostaglandin E_2_; 12-HETE, 12-Hydroxyeicosatetraenoicacid; CPD, Cyclobutane pyrimidine dimers; GTC = Green Tea Catechins.
